# Prediction of Postoperative Ambulatory Function Using Nomogram in Patients with Metastatic Epidural Spinal Cord Compression

**DOI:** 10.3390/jcm15197459

**Published:** 2026-09-25

**Authors:** Ibrahim S. Alayed, Sehan Park, Dong-Ho Lee, Chang Ju Hwang, Sung Tan Cho, Jae Hwan Cho

**Affiliations:** 1 Department of Spine Care, King Abdullah Medical City, Makkah 24246, Saudi Arabia; alayed.i2@kamc.med.sa; 2 Department of Orthopedic Surgery, Asan Medical Center, University of Ulsan College of Medicine, Seoul 05505, Republic of Korea; birdone86@amc.seoul.kr (S.P.); dlee@amc.seoul.kr (D.-H.L.); baski@amc.seoul.kr (C.J.H.); osdoctor0712@amc.seoul.kr (S.T.C.)

**Keywords:** metastatic epidural spinal cord compression, nomogram, postoperative ambulation, predictive model, decompressive surgery, neurological recovery, Karnofsky performance score, spinal metastasis

## Abstract

**Background/Objectives**: This study was conducted to develop and internally validate a nomogram that predicts postoperative ambulatory function in patients with metastatic epidural spinal cord compression (MESCC) undergoing decompressive surgery. **Methods**: Of 352 patients who underwent surgery for metastatic spinal cancer at a single tertiary center between 2014 and 2021, 97 with MESCC (Bilsky grade 2–3) and preoperative neurological deficits (ASIA grade D or worse) were included in this retrospective study. Binary logistic regression was used to identify candidate predictors of 3-month postoperative ambulatory status in the full cohort (*p* < 0.10 in univariable analysis), which were then entered into a multivariable logistic regression model and incorporated into a nomogram using the rms package in R. Model discrimination and calibration were internally validated using Harrell’s bootstrap optimism-correction method with 1000 resamples. **Results**: Postoperative ambulation was regained in 24 patients (24.7%). Four variables met the prespecified threshold (*p* < 0.10) for inclusion in the nomogram: preoperative motor grade (OR 2.48; 95% CI 1.41–4.34; *p* = 0.002), symptom onset–to–operation interval (OR 0.81; 95% CI 0.62–1.04; *p* = 0.098), preoperative KPS (OR 1.04; 95% CI 1.00–1.07; *p* = 0.070), and age (OR 0.97; 95% CI 0.93–1.01; *p* = 0.087); in the multivariable model, only preoperative motor grade and symptom onset-to-operation interval were independently associated with ambulatory recovery (*p* < 0.05). The nomogram achieved an apparent AUC of 0.827 (optimism-corrected, 0.800) and acceptable-to-good calibration (optimism-corrected calibration slope, 0.848; intercept, −0.088). At the Youden-optimal threshold, apparent (optimism-corrected) sensitivity, specificity, and F1 score were 79.2% (74.5%), 78.1% (76.7%), and 64.4% (60.6%), respectively. **Conclusions**: The nomogram demonstrated acceptable discrimination and good calibration for predicting postoperative ambulation in patients with MESCC. This tool may support individualized surgical decision-making and patient counseling. External validation is required before routine clinical use.

## 1. Introduction

Metastatic epidural spinal cord compression (MESCC) is estimated to affect approximately 5–10% of patients with cancer, with lung, breast, and prostate cancers representing the most common primary malignancies [1,2]. Recent advances in systemic therapies for patients with metastatic cancer have led to increased life expectancy [1,2]. However, patients with MESCC require active management to derive benefit from these treatments [1,2]. Although radiation therapy plays an important role in the treatment of MESCC, surgical decompression is required in patients presenting with neurological deficits to preserve ambulatory and motor functions [3]. Early diagnosis and intervention are critical for preserving neurological function and improving quality of life [4]. Recent surgical strategies for MESCC have increasingly applied the concept of “separation surgery,” in which tumor tissue adjacent to the spinal cord is removed to provide a safe margin for delivery of high-dose radiation [2,5]. Although stabilization with instrumentation may be applied on a case-by-case basis, meticulous separation of the spinal cord from tumor tissue often results in iatrogenic instability that warrants fixation [2,5]. This strategy, combined with postoperative radiation therapy, has improved the efficacy of surgery for MESCC while avoiding unnecessarily extensive operations that could increase patient morbidity and mortality [2].

Patients with MESCC require careful operative planning because of their complex clinical status, limited life expectancy, and increased risk of complications [5,6]. Surgical decision-making in this complication-prone population is often challenging. Some patients may experience substantial functional improvement after surgery, whereas others may experience deterioration in general condition or surgical complications [7,8]. Regaining ambulatory status after surgery may improve general performance status, quality of life, and willingness to pursue further systemic therapy [9,10]. Although individual prognostic factors associated with functional recovery have been reported, an integrated model predicting recovery of ambulatory status has not yet been reported [1].

Recent studies have reported integrated prognostic models, including predictive nomograms, scoring systems, and machine learning models [11,12]. Predictive nomograms offer the advantage of allowing surgeons to intuitively identify which factor plays a critical role in patient prognosis [11,12]. Furthermore, they may help identify modifiable factors associated with ambulatory recovery, thereby facilitating optimization of treatment strategies and improvement of outcomes.

Therefore, this study aimed to (1) develop a predictive nomogram to estimate the probability of postoperative ambulation after decompressive surgery for MESCC and (2) validate the predictive performance of the nomogram.

## 2. Materials and Methods

### 2.1. Study Design and Population

This retrospective study was approved by the Institutional Review Board of our institution (IRB 2024-0615). The requirement for informed consent was waived owing to the retrospective nature of the study. A total of 352 patients who underwent surgery for metastatic spinal cancer between March 2014 and March 2021 were retrospectively reviewed. The study period (2014–2021) was selected based on the completeness of clinical and follow-up documentation available for retrospective review. Patients were included if they (1) had MESCC with a Bilsky grade of 2 or 3 and (2) demonstrated preoperative neurological deficits classified as American Spinal Injury Association (ASIA) grade D or worse. Patients without preoperative neurological deficits or those who underwent surgery for pain alone were excluded [13]. (Figure 1).

All surgeries were performed at a single tertiary referral center by two attending spine surgeons (S.P. and J.H.C.) within the same spine team. No age-based exclusion criterion was applied. All patients had a pathologically confirmed diagnosis of the primary malignancy prior to spine surgery; the distribution of primary cancer types is presented in Table 1. The decision to operate was based on consensus reached at a multidisciplinary conference consisting of medical oncologists, spine surgeons, radiology oncologist, and rehabilitation specialist [1,2]. Patients’ life expectancy, performance status, symptoms, response to prior systemic therapy, and remaining treatment options were considered [2]. When multidisciplinary discussion was not feasible because urgent surgery was required, the decision to operate was made at the discretion of the attending spine surgeon [1]. Surgical strategies were individualized according to the location of metastasis, general health status, and spinal instability [2]. However, the principles of separation surgery, which require removal of tumor tissue surrounding the spinal cord to permit safe delivery of postoperative radiation therapy, were followed.

### 2.2. Outcome Variable

The primary outcome was postoperative ambulation. Patients who regained ambulatory function, with or without cane or walker assistance, were classified as ambulatory, whereas those who did not regain ambulatory function after surgery were classified as nonambulatory. Postoperative ambulatory status was assessed at 3 months after surgery or at the last follow-up for patients who survived <3 months postoperatively.

### 2.3. Predictor Variables

Perioperative variables, including demographic characteristics, surgical methods, Bilsky grade, Spine Instability Neoplastic Scores (SINS), individual components of the SINS, Karnofsky performance scores (KPS), preoperative motor grade, perioperative radiation therapy, symptom onset–to–operation interval, and preoperative embolization, were recorded. For motor strength assessment, the five key lower-extremity muscle groups defined by the American Spinal Injury Association (ASIA) scale (hip flexors [L2], knee extensors [L3], ankle dorsiflexors [L4], long toe extensors [L5], and ankle plantar flexors [S1]) were examined bilaterally using the standard manual muscle test (Medical Research Council scale, 0–5). The single highest grade among these ten bilateral muscle assessments was defined as the preoperative motor grade and used as the neurological predictor in subsequent analyses. This convention is consistent with our group’s previous work in this population [1] and is supported by evidence that individual key-muscle motor scores, rather than summed or averaged lower-extremity scores, can carry strong, independent prognostic value for ambulation recovery after spinal cord injury [1]. This variable is distinct from the ASIA Impairment Scale grade used for the inclusion criterion. Among the variables initially collected, candidate predictors were selected based on logistic regression analysis. Variables demonstrating a marginal association with postoperative ambulatory status (*p* < 0.10) in logistic regression analysis were included as explanatory variables in the nomogram [14].

Candidate variables meeting the univariable screening threshold (*p* < 0.10) were subsequently entered together into a single multivariable logistic regression model; this multivariable model, rather than the univariable estimates, forms the basis of the nomogram. Furthermore, classification performance was quantified using sensitivity, specificity, precision (positive predictive value), negative predictive value, and F1 score derived from a confusion matrix at a fixed probability threshold. The positive class was defined as regaining postoperative ambulatory function. The classification threshold was determined using the Youden index applied to the ROC curve of the model fitted on the full development cohort (threshold = 0.272); this fixed threshold was subsequently applied within each bootstrap resample to obtain optimism-corrected classification metrics. Sensitivity and recall represent the same quantity and are therefore reported as a single metric [15,16].

### 2.4. Statistical Analysis

Statistical analyses were performed using R software (version 4.4.2; R Foundation for Statistical Computing, Vienna, Austria). The outcome variable was postoperative ambulatory status (binary). Candidate predictor variables included age (continuous), the interval between symptom onset and operation (continuous), preoperative KPS (continuous), and preoperative motor grade (continuous). The rms package was used for regression modeling and nomogram construction, whereas the pROC and caret packages were used to assess model discrimination and classification performance [15].

The model was developed using the full cohort (*n* = 97). Internal validation was performed using bootstrap resampling with 1000 resamples, and optimism-corrected estimates of discrimination (C-index), calibration (slope and intercept), and classification performance (sensitivity, specificity, PPV, NPV, F1 score) were calculated using Harrell’s bias-correction method [16]. Both apparent (uncorrected) and optimism-corrected estimates are reported for each measure.

Regression coefficients from the final model were translated into a graphical nomogram to estimate individualized probability of postoperative ambulation. For graphical display, the axis for symptom onset–to–operation interval was truncated at 20 days, a range encompassing 97.9% of the cohort, to preserve visual resolution across the clinically relevant range; two patients with longer intervals (34 and 47 days) were retained in model fitting but are not depicted within the nomogram’s displayed range.

## 3. Results

### 3.1. Study Population Characteristics

Of the 352 patients who underwent surgery for metastatic spinal cancer during the study period, 71 were excluded because of Bilsky grade 0 or 1. Among the remaining 281 patients with Bilsky grade 2 or 3, 184 were excluded because they had no preoperative neurologic deficit and underwent surgery for pain alone or as a prophylactic procedure. The final analytic cohort therefore comprised 97 patients who were non-ambulatory before surgery (mean age, 59.1 ± 12.6 years; 58 men [59.8%]). The mean survival period after surgery was 12.4 ± 15.2 months. The most common primary pathology was lung cancer (24 patients, 24.7%) (Table 1). After surgery, 24 patients (24.7%) regained ambulatory function. The motor grade improved from 2.7 ± 1.4 preoperatively to 3.3 ± 1.3 postoperatively. Decompression combined with instrumentation was the most frequently performed surgical procedure (69.1%), and the mean number of operated levels was 1.9 ± 1.4 (Table 2).

Of 352 patients who underwent surgery for metastatic spinal cancer between March 2014 and March 2021, 71 were excluded because of Bilsky grade 0 or 1. Among the remaining 281 patients with Bilsky grade 2–3 spinal epidural compression, 184 were excluded because they had no preoperative neurologic deficit or underwent surgery for pain alone or as a prophylactic procedure. The final analytic cohort comprised 97 patients with preoperative neurologic deficit (ASIA grade D or worse) included in the analysis.

### 3.2. Factors Associated with Postoperative Ambulation and Nomogram Development

Twenty-four of 97 patients (24.7%) regained ambulatory function, corresponding to an events-per-variable ratio of 6 for the four candidate predictors. In binary logistic regression analysis, age (odds ratio [OR], 0.969; 95% CI, 0.932–1.008; *p* = 0.087), preoperative KPS (OR, 1.035; 95% CI, 0.997–1.073; *p* = 0.070), preoperative motor grade (OR, 2.477; 95% CI, 1.414–4.338; *p* = 0.002), and the interval between symptom onset and operation (OR, 0.805; 95% CI, 0.622–1.041; *p* = 0.098) demonstrated a marginal association with postoperative ambulation (Table 3). These four variables were subsequently entered into a multivariable logistic regression model (Table 4). In this model, preoperative motor grade (*p* = 0.001) and symptom onset-to-operation interval (*p* = 0.009) remained independently associated with postoperative ambulation, whereas age (*p* = 0.071) and preoperative KPS (*p* = 0.322) did not reach statistical significance.

A nomogram was developed based on these variables (Figure 2). The interval between symptom onset and operation contributed the greatest proportion of points, whereas preoperative KPS contributed the fewest points to the total score. To use the nomogram, points are assigned to each predictor by drawing a vertical line from the patient’s value to the “Points” axis; the points for all four predictors are then summed to obtain a “Total Points” score, which is projected onto the bottom axis to yield the predicted probability of postoperative ambulatory recovery.

The axis for symptom onset-to-operation interval is displayed over a 0–20-day range, representing 95 of 97 patients (97.9%) in the cohort. Two patients with intervals of 34 and 47 days were included in model fitting but fall outside the displayed axis range; their predicted probabilities were calculated directly from the regression equation rather than read from the nomogram.

### 3.3. Performance of the Predictive Nomogram

The ROC curve demonstrating the predictive performance of the nomogram is shown in Figure 3. The apparent AUC was 0.827 (optimism-corrected, 0.800). The ROC curve plots sensitivity against 1—specificity across all possible probability thresholds; an apparent AUC of 0.827 indicates that, in 82.7% of randomly selected ambulatory/non-ambulatory patient pairs, the model assigned a higher predicted probability to the patient who actually regained ambulation. The calibration plot (Figure 4) compares the nomogram’s predicted probabilities (*x*-axis) with the observed proportions of ambulatory recovery (*y*-axis); the apparent calibration slope and intercept were 1.00 and 0.00, and the optimism-corrected calibration slope and intercept were 0.848 and −0.088, respectively, indicating close agreement between predicted and observed outcomes, with a mild tendency toward overprediction once optimism was taken into account. At the Youden-optimal threshold of 0.272 (positive class: regained ambulation), the confusion matrix comprised 19 true positives, 57 true negatives, 16 false positives, and 5 false negatives. Apparent (optimism-corrected) sensitivity (=recall), specificity, precision, and F1 score were 79.2% (74.5%), 78.1% (76.7%), 54.3% (51.7%), and 64.4% (60.6%), respectively (Table 5).

### 3.4. Sensitivity Analysis

Of the 97 patients, 19 (19.6%) had ambulatory status ascertained before 3 months: 15 died within 1–2 months after surgery (all nonambulatory at last assessment) and 4 were alive with follow-up shorter than 3 months at the time of data extraction. A sensitivity analysis restricted to the 78 patients with documented ≥3-month follow-up yielded materially unchanged predictor estimates (motor grade OR 2.12, *p* = 0.004; symptom onset-operation interval OR 0.63, *p* = 0.011; age OR 0.96, *p* = 0.081; KPS OR 1.02, *p* = 0.442) and similar discrimination (apparent AUC 0.819 vs. 0.827 in the full cohort), suggesting that inclusion of early-ascertained outcomes did not introduce substantial bias (Supplementary Table S1).

## 4. Discussion

In this study, a nomogram for predicting postoperative ambulatory function in patients with MESCC was developed and internally validated. Logistic regression analysis identified four candidate predictors that met the prespecified screening threshold (*p* < 0.10) and were incorporated into the nomogram: age, preoperative KPS, preoperative motor grade, and the interval between symptom onset and surgical intervention; among these, only preoperative motor grade and symptom onset-to-operation interval were independently associated with ambulatory recovery in the multivariable model (Table 4) at the conventional *p* < 0.05 threshold. The resulting nomogram demonstrated acceptable-to-good discrimination (apparent AUC, 0.827; optimism-corrected AUC, 0.800; conventionally classified as 0.7–0.8, acceptable, to 0.8–0.9, excellent) [16], and acceptable-to-good calibration based on bootstrap resampling. At its current stage of development, this nomogram should be regarded as exploratory and hypothesis-generating rather than ready for direct clinical application, given the single-center retrospective design, limited number of outcome events, and absence of external validation.

Ambulatory status after surgery is one of the most clinically meaningful outcomes in patients with MESCC [1,17]. The ability to walk independently is strongly associated with improved quality of life and longer survival, whereas loss of ambulation is a significant predictor of functional dependence and reduced life expectancy [18]. Despite this, predicting which patients will recover ambulatory function after surgical decompression remains challenging [2]. Previous studies have tended to analyze individual prognostic factors in isolation, without providing a tool that integrates multiple variables into a single, clinician-accessible prediction score [19,20]. The nomogram developed in this study addresses this gap by combining four clinically relevant and readily available variables into a graphical model that generates individualized probability estimates of postoperative ambulation. Quantification of risk in this manner may assist surgeons, oncologists, and rehabilitation specialists in engaging patients and families in more informed and goal-aligned discussions regarding the potential benefits and risks of surgical intervention [21,22].

Among the four predictors included in the nomogram, preoperative motor grade showed the strongest association with postoperative ambulation (OR, 2.477). This finding is consistent with the existing literature. Several studies have demonstrated that preserved preoperative motor function is one of the strongest predictors of neurological recovery after decompressive surgery in patients with MESCC [19,23]. The biological rationale is that patients with better residual motor function are likely to have less extensive spinal cord injury, thereby preserving greater potential for neurological recovery following surgical decompression [1]. Furthermore, higher preoperative motor grades are associated with earlier postoperative ambulation, which may facilitate rehabilitation and reduce complications related to immobility [8,17].

The interval between symptom onset and surgical intervention was independently associated with postoperative ambulation in the revised multivariable model (OR, 0.648; 95% CI, 0.469–0.896; *p* = 0.009; Table 4). However, as this is an observational, retrospective, single-center study, this association cannot establish that shortening this interval would itself causally improve the probability of ambulatory recovery; unmeasured factors related to disease biology, symptom severity, or referral patterns may also contribute to this association. The importance of timely surgical intervention in MESCC is nonetheless well supported by prior evidence. Earlier surgery has consistently been associated with better neurological outcomes in previous studies, with a proposed pathophysiological mechanism involving progressive ischemia and irreversible axonal injury resulting from sustained spinal cord compression [20]. Wänman et al. reported that patients who had been unable to walk for more than 48 h before surgery experienced significantly lower rates of ambulation recovery [23]. Taken together with this existing body of evidence, our findings are consistent with, rather than independently establishing, the clinical principle that delays in referral or operative planning should be minimized in patients with new or progressive neurological deficits attributable to MESCC.

Preoperative KPS was included as a marker of overall functional reserve and performance status. Patients with higher KPS are more likely to demonstrate greater physiological resilience and to tolerate the demands of surgery and subsequent rehabilitation. In the context of spinal metastases, KPS has been validated as a predictor of both survival and postoperative functional outcomes across multiple prognostic scoring systems, including the revised Tokuhashi and Tomita scores [24,25,26]. Although the OR for KPS in the present study was modest (1.035 per unit; *p* = 0.070), its inclusion in the integrated model is supported by its additive predictive value in combination with the other variables and by the prespecified significance threshold of *p* < 0.10 for variable selection.

Age demonstrated a trend toward a reduced probability of postoperative ambulation (OR, 0.969 per year; *p* = 0.087), suggesting that older patients may have diminished neurological plasticity, reduced physiological reserve, and a greater burden of comorbidities that can impair rehabilitation and recovery. While age alone is not a contraindication to surgery, it represents an important contextual factor in the overall risk-benefit assessment of surgical intervention for MESCC. Integration of age into the nomogram allows clinicians to account for this biological factor alongside disease-specific and neurological variables when counseling patients [8].

Should future studies confirm the performance of this nomogram in external cohorts, several potential clinical applications may be envisioned. First, it could serve as an adjunct during preoperative counseling, providing individualized, data-driven probability estimates to complement—rather than replace—clinical judgment when discussing the likelihood of regaining ambulatory function with patients and families [27]. This could be particularly relevant in MESCC, where decisions regarding surgery are often complex, time-sensitive, and emotionally challenging. Second, the nomogram could potentially inform multidisciplinary tumor board discussions, in which clinicians from oncology, radiation oncology, neurosurgery, and rehabilitation medicine collectively weigh the potential functional benefits of surgery against its risks in the context of a limited prognosis. Third, it might eventually assist in stratifying patients according to anticipated postoperative rehabilitation needs [28]. We emphasize, however, that none of these applications is appropriate for current clinical practice without prospective external validation, consistent with the exploratory, hypothesis-generating nature of this model at its present stage of development.

The model demonstrated an apparent AUC of 0.827 (optimism-corrected, 0.800), indicating acceptable-to-good discriminative ability. At the Youden-optimal threshold, apparent sensitivity and specificity were similar in magnitude (79.2% and 78.1%, respectively; optimism-corrected, 74.5% and 76.7%). Overprediction is properly assessed through calibration rather than through a comparison of sensitivity and specificity at a single threshold; the optimism-corrected calibration intercept was −0.088 and the calibration slope was 0.848, indicating a mild tendency of the model to overpredict the probability of ambulatory recovery on average (calibration-in-the-large) and a modest degree of overfitting (probability estimates slightly more extreme than the data support), respectively, once optimism was taken into account. The apparent Brier score was 0.139 (optimism-corrected, 0.158). These findings indicate that, while discrimination and calibration were acceptable in the development cohort, some degree of optimism is expected when the model is applied to new patients, and the current probability estimates should be interpreted with appropriate caution rather than assumed to be reliable at the individual patient level pending external validation.

A threshold of *p* < 0.10 was applied for variable selection in the logistic regression model. Although a *p*-value of 0.05 is the conventional threshold for statistical significance, the use of a more liberal threshold of 0.10 is widely accepted in the development of multivariable clinical prediction models, particularly when the objective is to identify variables with potential predictive relevance rather than to establish causal inference. This approach reduces the risk of excluding clinically important variables because of type II error, particularly in studies with limited sample sizes. Hosmer and Lemeshow have advocated the use of a *p*-value threshold of ≤0.25 for initial variable screening in logistic regression, and many published prediction model studies have employed thresholds ranging from 0.10 to 0.20 [14]. Given the sample size of 97 patients in the present study, the use of a 0.10 threshold was considered appropriate to minimize premature exclusion of clinically plausible predictors [14].

Several limitations should be acknowledged. First, the retrospective design introduces potential selection bias and limits control over unmeasured confounders. Second, the sample size of 97 patients is relatively small, which may have constrained the statistical power of the logistic regression analysis and contributed to the suboptimal discriminative performance of the model. A larger prospective cohort would enable more robust variable selection and model development. Third, internal validation was performed using bootstrap resampling of the full development cohort; external validation in an independent, geographically distinct cohort is still required before this nomogram can be recommended for routine clinical use. Fourth, several variables that may influence postoperative ambulation, such as primary tumor histology, presence of visceral metastases, systemic treatment regimen, and postoperative rehabilitation intensity, were not included in the final model, either due to limited statistical association or concerns regarding overfitting given the sample size. Future studies incorporating larger multicenter datasets and more comprehensive variable sets may further improve predictive accuracy. Fifth, the events-per-variable ratio in this cohort (6 events per predictor) is below the traditionally recommended threshold of 10 [29], which may limit the precision of individual coefficient estimates. However, bootstrap internal validation indicated only modest optimism in model discrimination (C-index optimism = 0.027), suggesting that overfitting did not substantially inflate the reported performance. Sixth, the use of ambulation at three months as the primary outcome, although clinically relevant, may not capture longer-term functional trajectories. Future investigations should consider assessing ambulation at multiple time points [22,30]. Finally, preoperative motor grade was defined as the single highest grade among ten bilateral key lower-extremity muscle assessments; although this convention is consistent with our group’s prior work in this population [1] and is supported by evidence that individual key-muscle strength—rather than a summed or averaged score—can be strongly and independently prognostic for ambulation [31,32], this approach may not fully capture the severity of impairment in patients with asymmetric or multifocal weakness, and alternative aggregation methods (e.g., a summed motor score) were not evaluated in the present study.

## 5. Conclusions

A nomogram was developed and internally validated to predict postoperative ambulatory function in patients with MESCC undergoing decompressive surgery. The model incorporates age, preoperative KPS, preoperative motor grade, and the interval from symptom onset to surgery, and demonstrated acceptable discrimination and good calibration. This tool may assist clinicians in individualizing surgical decision-making and facilitating informed, patient-centered discussions. External validation in larger, multicenter cohorts is warranted to confirm generalizability and support integration into routine clinical practice.

## Figures and Tables

**Figure 1:**
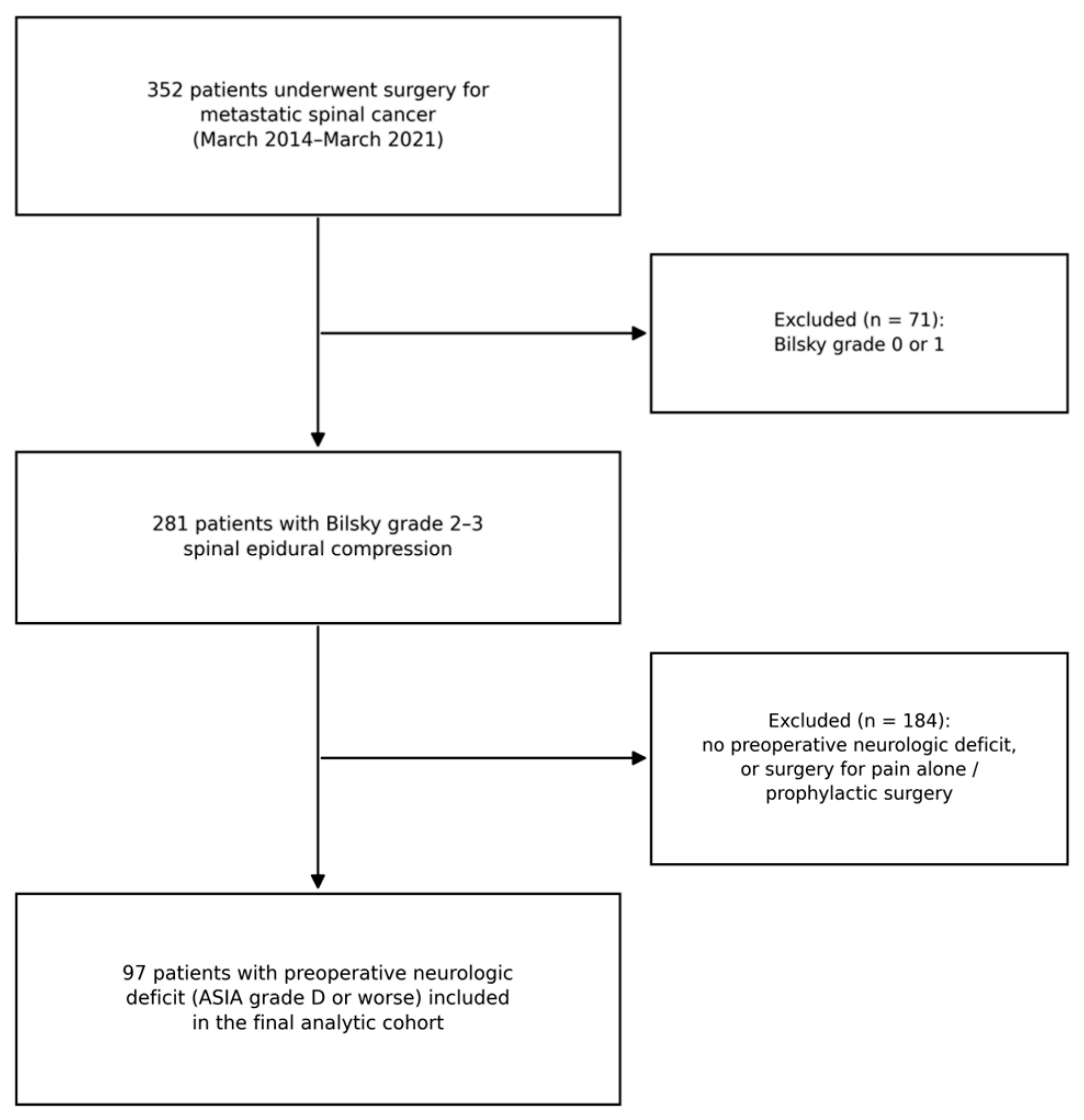
Flow diagram of patient selection.

**Figure 2:**
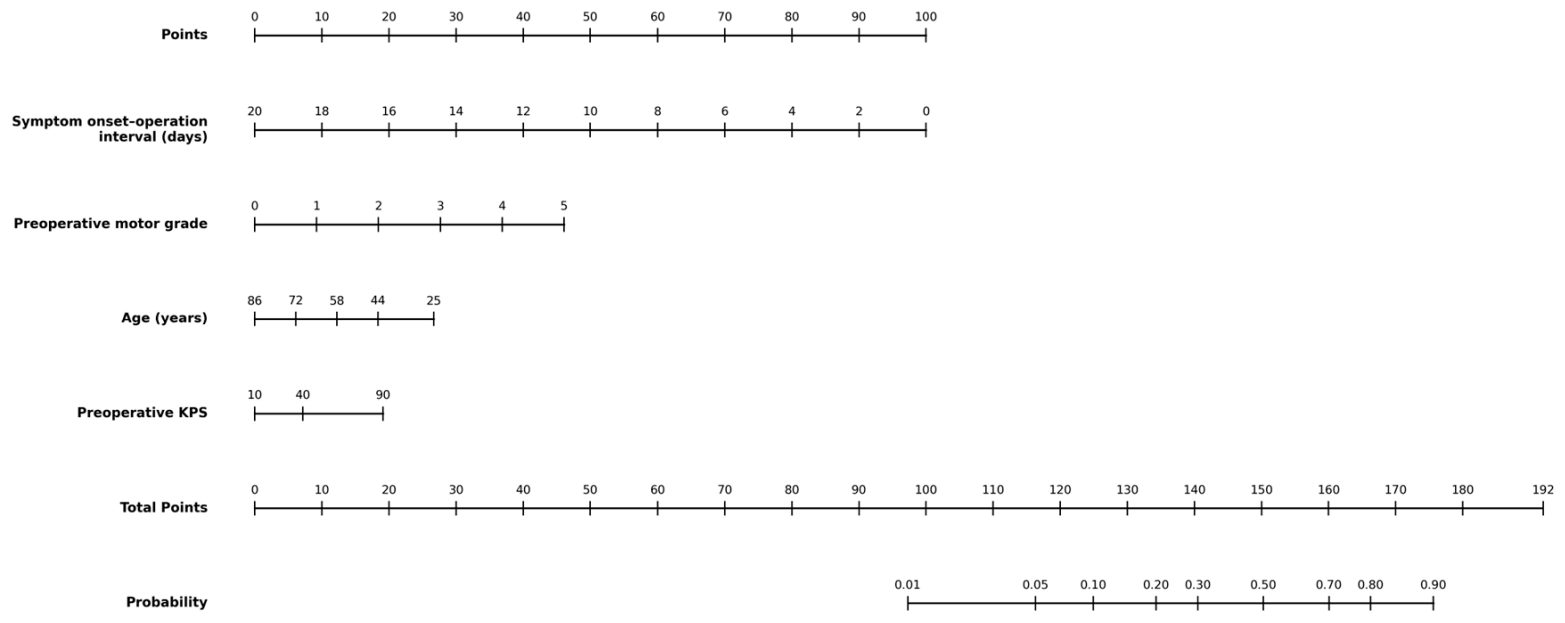
Nomogram for predicting postoperative ambulatory function.

**Figure 3:**
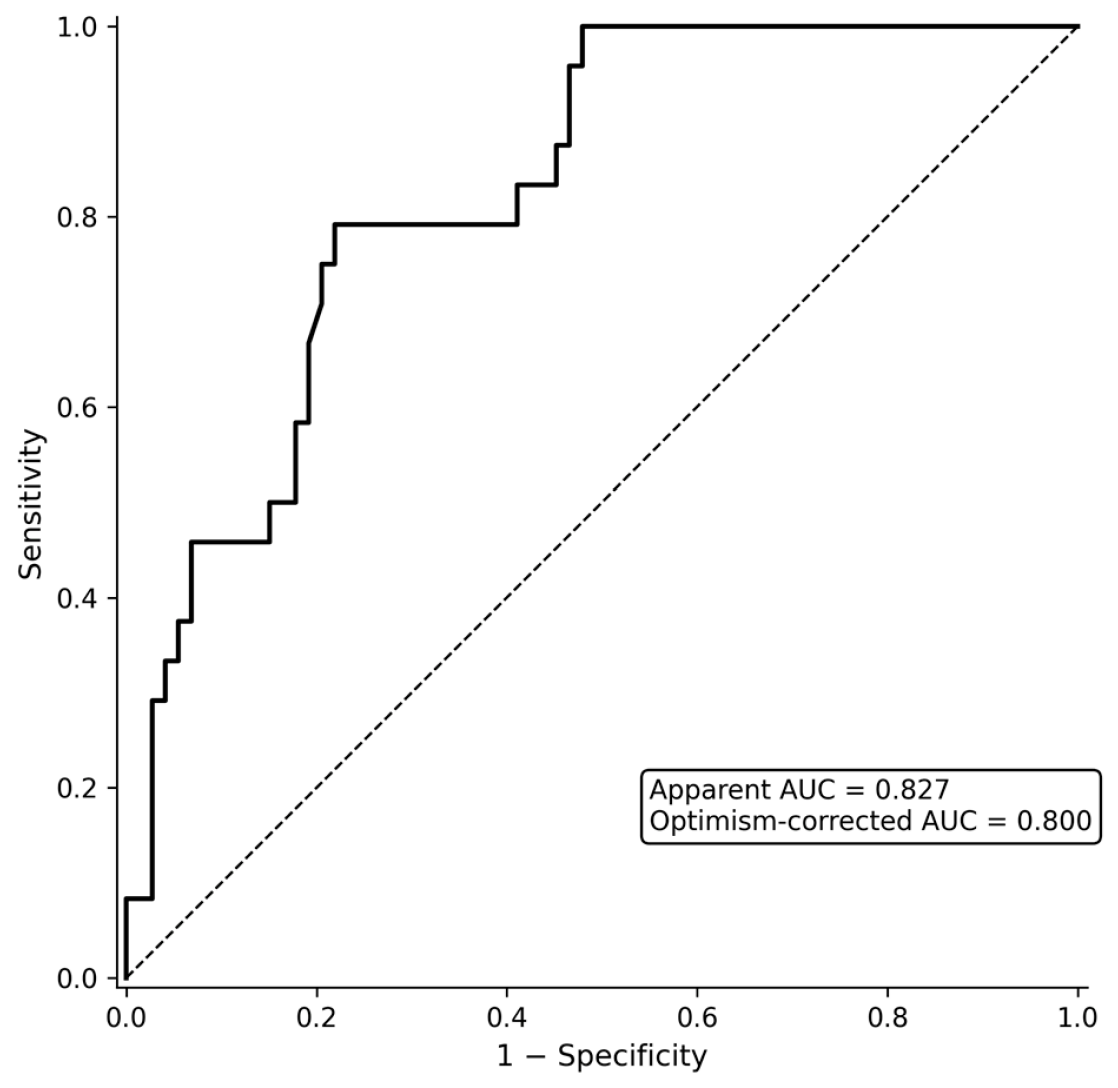
Receiver operating characteristic curve.

**Figure 4:**
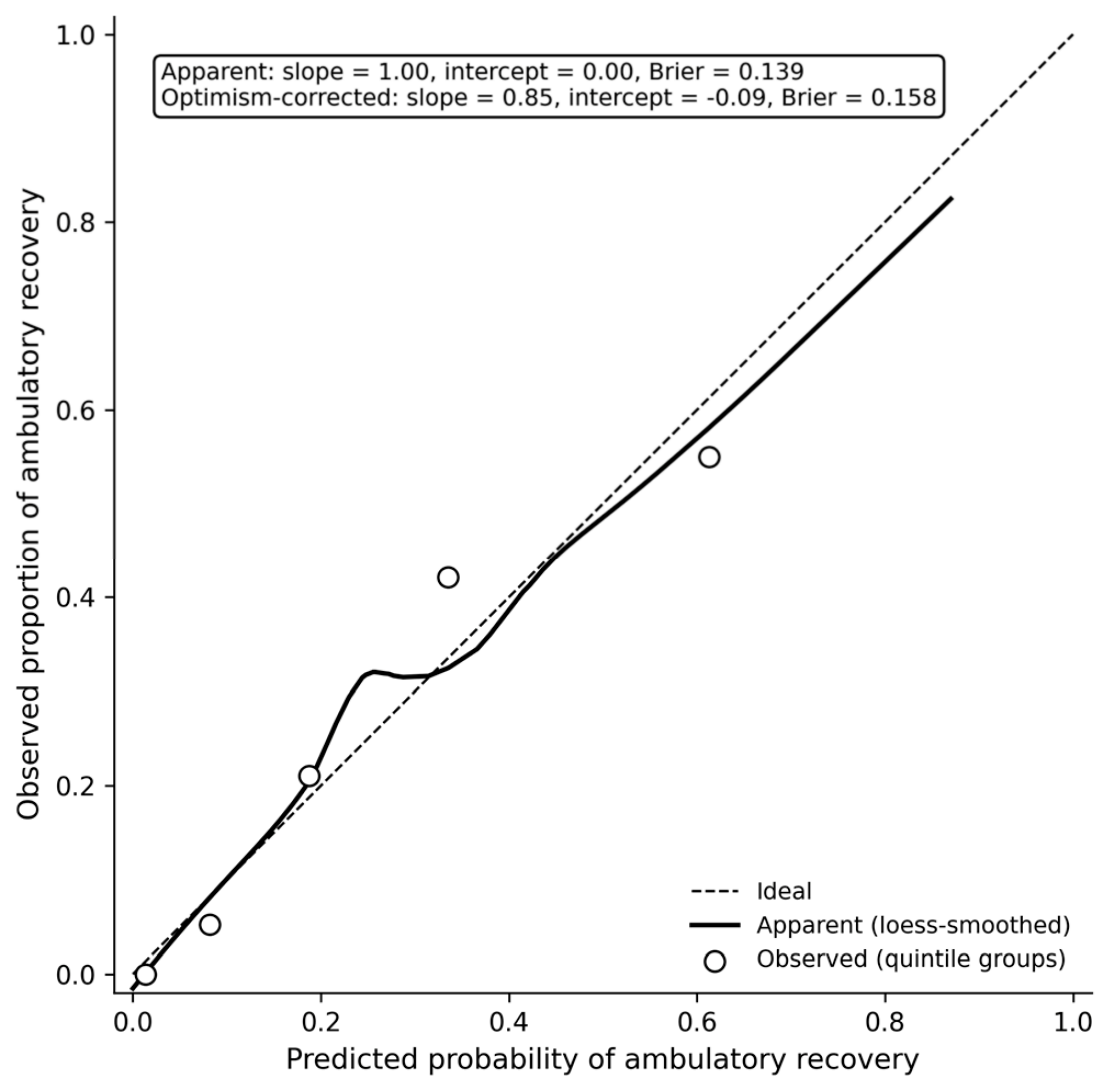
Calibration curve.

**Table 1:** Baseline characteristics of the study population.

Factors	Value
Age	59.1 ± 12.6 (range, 25–86)
Sex (Male:Female)	58:39
Body mass index	23.5 ± 5.1
Survival (months)	12.4 ± 15.2
Comorbidities	
Diabetes mellitus	21 (21.6%)
Hypertension	31 (32.0%)
Major organ metastasis	
None	65 (67.0%)
Resectable	2 (2.1%)
Nonresectable	30 (30.9%)
Primary cancer	
Lung	24 (24.7%)
Breast	5 (5.2%)
Prostate	11 (11.3%)
Renal	5 (5.2%)
Thyroid	1 (1.0%)
Hepatic	0 (0.0%)
Stomach, colon	5 (5.2%)
Others	46 (47.4%)

**Table 2:** Functional, oncologic, and treatment-related factors.

Factors	Value
Motor grade	
Preop	2.7 ± 1.4
Postop	3.3 ± 1.3
Postop ambulation	24 (24.7%)
KPS score (preop)	49.8 ± 13.3
RT	
Preop	37 (38.1%)
Postop	49 (50.5%)
Op procedures	
Decompression only	8 (8.2%)
Fixation only	2 (2.1%)
Decompression and fixation	67 (69.1%)
Corpectomy or en bloc resection	20 (20.6%)
Number of operated levels	1.9 ± 1.4
Neoplastic factors	
Anterior dural compression	92 (94.8%)
Posterolateral involvement	87 (89.7%)
Pathologic VCF	74 (76.3%)
Bilsky grade	2.6 ± 0.6
SINS	10.4 ± 3.0

KPS, Karnofsky performance score; RT, radiation therapy; VCF, vertebral compression fracture; SINS, spinal instability neoplastic score.

**Table 3:** Logistic regression analysis of factors associated with postoperative ambulatory recovery.

	Odds Ratio	95% Confidence Interval	*p* Value
Demographic factors			
Age	0.969	0.932–1.008	0.087
Sex	1.760	0.653–4.744	0.264
BMI	0.892	0.764–1.042	0.149
KPS score (preop)	1.035	0.997–1.073	0.070
Motor grade (preop)	2.477	1.414–4.338	0.002 *
Neoplastic factors			
Number of levels operated	1.151	0.816–1.624	0.421
Anterior dural compression	0.596	0.160–2.226	0.442
Posterolateral involvement	1.147	0.219–5.998	0.871
Pathologic VCF	2.667	0.705–10.092	0.149
Bilsky grade	0.613	0.281–1.339	0.220
SINS	1.044	0.888–1.228	0.600
Treatment-related factors			
Operation method			
Decompression only	n/a	n/a	n/a
Fixation only	n/a	n/a	n/a
Decompression and fixation	2.298	0.603–8.754	0.223
Corpectomy or en bloc resection	0.736	0.186–2.907	0.662
Preop RT	1.370	0.502–3.733	0.539
Postop RT	2.017	0.737–5.524	0.172
Symptom onset to operation interval	0.805	0.622–1.041	0.098
Preoperative embolization	0.703	0.226–2.185	0.543

BMI, body mass index; KPS, Karnofsky performance score; VCF, vertebral compression fracture; SINS, spinal instability neoplastic score; RT, radiation therapy. * *p* < 0.05.

**Table 4:** Multivariable logistic regression model for postoperative ambulatory recovery.

Variable	Coefficient	SE	OR	95% CI	*p*
Intercept	−1.306	1.709	—	—	0.445
Age	−0.038	0.021	0.963	0.924–1.003	0.071
Symptom onset–operation interval	−0.433	0.165	0.648	0.469–0.896	0.009 *
Preoperative KPS	0.021	0.021	1.021	0.980–1.064	0.322
Preoperative motor grade	0.799	0.245	2.223	1.375–3.595	0.001 *

SE, standard error; OR, odds ratio; CI, confidence interval; KPS, Karnofsky performance score. * *p* < 0.05.

**Table 5:** Performance metrics of the predictive nomogram (apparent and optimism-corrected).

Metric	Apparent	Optimism-Corrected
AUC	0.827	0.800
Sensitivity (=Recall)	79.2%	74.5%
Specificity	78.1%	76.7%
Precision (PPV)	54.3%	51.7%
NPV	91.9%	89.9%
F1 score	64.4%	60.6%

AUC, area under the curve; PPV, positive predictive value; NPV, negative predictive value. Classification threshold = 0.272 (Youden index, applied to the ROC curve of the model fitted on the full cohort, *n* = 97). Positive class: regained postoperative ambulatory function. Confusion matrix at this threshold (apparent): true positive = 19, true negative = 57, false positive = 16, false negative = 5. Optimism-corrected estimates were obtained by applying this fixed threshold within each of 1000 bootstrap resamples.

## Data Availability

The data presented in this study are available from the corresponding author upon reasonable request.

## Supplementary Materials

The following supporting information can be downloaded at: https://www.mdpi.com/article/10.3390/jcm15197459/s1, Supplementary Table S1. Sensitivity analysis excluding patients with limited postoperative outcome ascertainment.
