# Supplementary material for: Prediction of Postoperative Ambulatory Function Using Nomogram in Patients with Metastatic Epidural Spinal Cord Compression
Source: J Clin Med. 2026 Sep 25;15(19):7459. doi: 10.3390/jcm15197459 (PMC13666026; doi:10.3390/jcm15197459)
Supplement: Supplementary file 1 [file jcm-15-07459-s001.zip › jcm-4552791-supplementary.pdf]

Supplementary Table S1. Sensitivity analysis excluding patients with limited postoperative outcome ascertainment.

Variable	Original cohort (n = 97)<br>OR (95% CI), p	Sensitivity cohort (n = 78)*<br>OR (95% CI), p
Age	0.963 (0.924–1.003), 0.071	0.963 (0.922–1.005), 0.081
Symptom onset–operation interval	0.648 (0.469–0.896), 0.009 †	0.631 (0.442–0.900), 0.011 †
Preoperative KPS	1.021 (0.980–1.064), 0.322	1.019 (0.972–1.067), 0.442
Preoperative motor grade	2.223 (1.375–3.595), 0.001 †	2.124 (1.277–3.534), 0.004 †
Apparent AUC	0.827	0.819

OR, odds ratio; CI, confidence interval; KPS, Karnofsky performance score; AUC, area under the receiver operating characteristic curve.

* The sensitivity cohort excludes 19 patients with limited outcome ascertainment (survival < 3 months from surgery: 15 who died before 3 months, all of whom had not regained ambulation, and 4 who were alive but had less than 3 months of follow-up at last contact). Coefficient direction, magnitude, and statistical significance were materially unchanged, supporting the robustness of the primary model (n = 97) to this potential source of outcome misclassification.

† $p < 0.05$.
